# Benefits of Motion in Animated Storybooks for Children’s Visual Attention and Story Comprehension. An Eye-Tracking Study

**DOI:** 10.3389/fpsyg.2016.01591

**Published:** 2016-10-13

**Authors:** Zsofia K. Takacs, Adriana G. Bus

**Affiliations:** ^1^Institute of Education, Faculty of Education and Psychology, Eötvös Loránd UniversityBudapest, Hungary; ^2^Learning Problems and Impairments, Institute of Education and Child Studies, Leiden UniversityLeiden, Netherlands

**Keywords:** multimedia learning, electronic storybooks, animation, motion, story comprehension, vocabulary learning, eye-tracking, attention

## Abstract

The present study provides experimental evidence regarding 4–6-year-old children’s visual processing of animated versus static illustrations in storybooks. Thirty nine participants listened to an animated and a static book, both three times, while eye movements were registered with an eye-tracker. Outcomes corroborate the hypothesis that specifically motion is what attracts children’s attention while looking at illustrations. It is proposed that animated illustrations that are well matched to the text of the story guide children to those parts of the illustration that are important for understanding the story. This may explain why animated books resulted in better comprehension than static books.

## Introduction

In contrast to concerns expressed in the literature for a “mesmerizing” effect of computers and screens on young children’s cognitive development (e.g., Hayes and Birnbaum, 1980; Spitzer, 2012), there is nevertheless growing evidence for the potential of new formats enabled by computer technology for fostering children’s learning. In line with Mayer’s (2003) multimedia learning theory it seems helpful to young children when narrative text is accompanied by illustrations just as graphics are helpful when they are added to informative text. When information from different sources are simultaneously available it supports the integration of images and language, and the verbal information will be understood and retained better than if conveyed by words alone (Paivio, 2007). Reviews focusing on software for young children have shown that, apart from static pictures, in particular animated pictures can be helpful additions to stories (Kamil et al., 2000; Zucker et al., 2009; Van Daal and Sandvik, 2011). If electronic books contain animated pictures this supports learning even in the absence of parental mediation (Strouse et al., 2013). The aim of the current study was to specify why motion pictures in electronic books may provide guidance to the young learner, more so than printed books with only static illustrations.

When children began to spend a constantly increasing amount of time watching television educators were afraid that the motion pictures would distract children’s attention from the language and thus interfere with story comprehension and learning new vocabulary. So far there is not much empirical evidence for the so-called visual superiority hypothesis, proposing that looking at motion pictures is more appealing to young children than listening to the oral language (Hayes and Birnbaum, 1980). When the two sources of information – narration and motion pictures – were not mismatched, the initial research finding of a negative effect of motion pictures on language recall disappeared in line with the dual coding theory (Pezdek and Stevens, 1984; Pezdek et al., 1984; Rolandelli, 1989). According to dual coding theory, the two kinds of stimuli, non-verbal information and narration, can be processed simultaneously without causing cognitive overload. They are processed in separate but interconnected channels thus enhancing mental representations and memory traces that connect details of pictures with phrases in the narrative (Paivio, 2007).

Recent studies show that motion pictures built in storybooks make picture storybook exposure – one of the strongest incentives for developing language and literacy skills in the preschool age (Snow et al., 1998) – even more effective (e.g., Ricci and Beal, 2002; Segers et al., 2006; Verhallen et al., 2006; Segal-Drori et al., 2010; Homer et al., 2014). It is apparent from the literature that multimedia features in the new electronic formats of picture storybooks, as long as those additions are well matched to the verbal narration, boost the effects of stories on young children’s story comprehension and word learning (for meta-analytic evidence see Takacs et al., 2015). These findings may have great practical relevance and may improve the format of the increasingly growing supply of multimedia storybooks in App stores.

When we designed this study we were specifically interested in explaining why motion pictures are more beneficial for young children’s story comprehension than just static pictures (Takacs et al., 2015). Based on prior studies using eye-gaze methodology we hypothesized that, due to motion, children’s eyes may focus on those parts of the illustration that are highlighted by the text and thus help children to connect words to visual information and cement these associations firmly in memory. Prior studies have shown that eye fixations are time locked to referential expressions in the text which evidences that children integrate the images and the language. Evans and Saint-Aubin (2005) showed that fixations on details in illustrations can be changed by altering the content of the text. Eye fixations on small details in the illustrations (a fish, a boat, or two stars) increased when the text highlighted those details, thus demonstrating that the children’s visual attention was dependent on the accompanying text. In the same vein, Verhallen and Bus (2011) found that visual elements that the text highlighted were fixated more often and longer than elements in illustrations similar in color, size and other characteristics but not highlighted in the story text. Adult support during book reading implies that they, by pointing and commenting, guide children’s visual attention to elements of the illustration that are highlighted in the text but due to unfamiliar language not recognized by young children (Strouse et al., 2013) thus enforcing the integration of verbal and non-verbal information. In the current study we tested whether motion provides similar support by guiding children’s attention to details of the illustrations that are simultaneously highlighted in the narration and thus helps children to integrate picture and narration.

**FIGURE 1:**
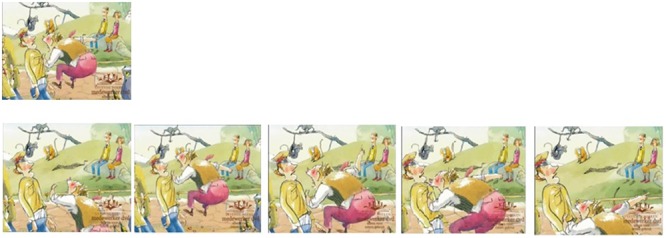
**One of the target illustrations chosen for fine-grained analysis of the eye-tracking data.** The same illustration in the static condition in the first row and still frames from the animated version are shown in the second row. The director of the zoo is jumping up and down while the accompanying oral text says: ‘These are not monkeys!’ he shouted ^∗^jumping^∗^ ‘These are people!’. [‘Dit zijn geen apen!’ riep hij “trinnend,” ‘Dit zijn mensen!’. Copyright 2014 by Het Woeste Woud.

The current study aimed at testing the effect that motion in illustrations has on children’s eye movements in explanation of the benefits that animated books have on integrating non-verbal information and narration to support story comprehension and exposure to unknown words. Children are known to be especially attentive to rapid action (Potts et al., 1986), animation, and motion (Levin and Anderson, 1976; Alwitt et al., 1980). This study tests the hypothesis that motion can be an effective tool to guide children’s visual attention to details in the picture that are highlighted by the text resulting in more visual attention to those details than during reading a book with just still pictures. It is also possible that motion helps children to target a detail in an illustration instead of scanning the whole picture resulting in longer average fixations on the target detail. Motion in an illustration might thus help to concretize the story language that children simultaneously hear which supports understanding the story and specific words in the text. For example, in **Figure 1** the angrily looking director is the only part of the illustration in motion, thereby probably attracting the most attention despite many other visualized story details in the picture. Fixating this detail in the illustration may help to comprehend the simultaneously spoken complex text fragment saying: “He shouted jumping.” In testing the theory that motion attracts attention we contrasted effects of listening to stories with still and animated pictures. A confounding factor might be that motion implies information that is not available in the static version of the book. We were therefore careful to select still equivalents for the animated fragments that provide exactly the same information. For example, in the scene in **Figure 1**, the motion in the animated version (see the second row in **Figure 1**) depicts the meaning of “He shouted jumping.” As a still picture we selected a representative stop motion frame from the animated segment that implied the same motion (see the first row in **Figure 1**). There cannot be any misunderstanding that the director in the still picture is jumping: the director is depicted with his feet in the air.

It is also possible that children’s overall attentiveness when listening to stories is higher with animated as compared to static illustrations (Verhallen and Bus, 2009; Moody et al., 2010). There is some evidence showing that young children seem to prefer a multimedia presentation of stories over a static presentation as in print books and are more attentive to animated materials. Verhallen and Bus (2009) found that skin conductance – an indicator of children’s mental effort during listening to storybooks (e.g., Pecchinenda and Smith, 1996) - remained at the same level over four repeated readings of a story when the book included multimedia features like motion pictures, music and sound effects. However, mental effort decreased in the third and fourth repetition when the same story included only static illustrations. In the same vein, Moody et al. (2010) revealed that children were more persistent when sharing an electronic storybook as compared to sharing a traditional print storybook with an adult. The present study investigated whether there is an overall elevated visual attention when listening to animated versus static books, that is, whether animated illustrations attract more visual attention than their static counterparts.

In the present study children repeatedly listened to two stories: one with static pictures and another with animated illustrations. Both books were presented three times on an eye-tracker. We utilized eye-tracker data two ways: first, to register children’s overall visual attention to the illustrations when listening to animated or static stories. Secondly, in order to test whether motion in illustrations indeed attracted children’s attention more than the same details in static illustrations, we selected three pages per book with a detail in motion like in **Figure 1** and compared visual attention for this detail with attention for the same detail in the static version of the book. To the best of our knowledge, this was the first study comparing children’s visual attention to animated and static details in pictures. We examined, apart from children’s recall of the story language, their learning of new words as a function of animated versus static pictures. Previous studies have shown that differences in experiences with words strongly vary across children and make it hard to determine how much learning resulted from repeated readings of a particular book (e.g., Smeets and Bus, 2012). To maximally control for differences in word knowledge resulting from prior exposure to the target words we preferred adding non-words (that is, words that do not exist in the Dutch language but sound like Dutch) to each book replacing well-visualized words from the story text. This way we were sure that children did not have any previous knowledge of the target words.

### Hypotheses

(1) Based on the previous literature showing an advantage of multimedia-enhanced stories over print-like static stories (Takacs et al., 2015), we expected that children would recall more from the language and the content of the story when encountering stories with illustrations that include details in motion that are highlighted in the text as compared to still pictures that provide exactly the same information but without motion.

(2) In the same vein, animated illustrations may, more than static illustrations, facilitate the learning of the non-words in particular when details that relate to the non-words are in motion. Since the non-words were completely unknown we expected, as a first step toward learning these words, effects on receptive knowledge rather than on expressive word knowledge (Nagy and Scott, 2000; Smeets and Bus, 2012).

(3) Details in motion were expected to attract more visual attention than static details (Levin and Anderson, 1976; Alwitt et al., 1980). Accordingly, for the detail that is in motion we predicted longer total time of fixation as well as longer average fixations compared to exactly the same detail in a static illustration.

(4) It is also possible that children are more visually attentive to illustrations that include motion than to static illustrations (Verhallen and Bus, 2009; Moody et al., 2010) maybe because animated books might be more engaging. In this case screens in the animated condition may attract more visual attention than in the static condition.

## Materials and Methods

The study was approved by the ethical committee of the Institute of Education and Child Studies at Leiden University under the reference number ECPW-2012/044.

### Participants

Children were recruited from 5 kindergarten classrooms in 3 public schools with 4-, 5-, and 6-year-old children who had not yet received formal reading instruction. In the Netherlands formal reading instruction including intensive daily practice starts in grade 1. Two schools were located in sub-urban neighborhoods attracting a middle-class population; one school recruited children from a village attracting a more mixed population. Parents of 43 children gave informed consent for participation of their child in the study. All participants were according to the teacher typically developing and not delayed in language or literacy, this was the only criterion for inclusion in the study. Among the 43 children were three children who were excluded from the study because they were siblings of other participants. Also, one boy’s eye-tracking data collected at the second session was lost and he was excluded from all further analyses. The final sample consisted of 39 children (22 boys and 17 girls) with a mean age of 61.26 months (*SD* = 7.69, range: 48–77 months). From the three participating schools we recruited 9, 6, and 24 children, respectively.

### Design

The study was a within-subject design in which every child participated in three conditions: a storybook with animated illustrations, a storybook with static illustrations and a control condition, including only post-testing and no book reading. We decided to use a control condition because otherwise we cannot be sure that the quality of the retellings is the result of listening to the story instead of just seeing the pictures during the retelling. Note that the pictures visualized most story events. The illustrations in both the animated and static conditions were the same and presented for exactly the same amount of time going together with the same oral narration, the only difference being the presence of motion in the animated versions. As young children need repeated encounters with stories before it is possible to assess teach (Verhallen et al., 2006) the experimental stories were presented three times. Three storybooks can be assigned to three conditions in six different ways. The participants were about equally assigned to these six possibilities.

### Procedure

Sessions in which children listened to the stories took place at school in a spare room not in use for other activities. In all there were three sessions. In each session children listened to two stories, one in the animated format and the other in the static format. The animated version of a book was presented for the same amount of time as the static version of the same book. As shown in **Table 1**, two sessions took place on the first day and one on a second day, on average 2 days later (*M* = 2.00, *SD* = 1.12). In the third session children listened to the two books in the opposite order as in the previous sessions (as shown in **Table 1**). The researcher was present and started up the books. To register visual attention while hearing the narration the books were presented on the screen of an eye-tracker. Children sitting in front of the eye-tracker screen looked at the animated and static pictures while they listened to the narration by the computer voice.

**Table 1:** An example of the schedule of the experiment.

Day 1	Day 2
**Session 1**	**Session 3**
Reading of animated version of *Imitators*	Reading of static version of *The Little Kangaroo*
Reading of static version of *The Little Kangaroo*	Reading of animated version of *Imitators*
**Session 2**	**Post-testing**
Reading of animated version of *Imitators* Reading of static version of *The Little Kangaroo*	Retellings of the three stories *(1) The Bear is in Love with Butterfly* *(2) Imitators* *(3) The Little Kangaroo*
	Vocabulary tests: Expressive vocabulary test Context integration test Receptive vocabulary test Meaning recognition test

*There was 1–5 days between the two sessions. We used the three books in the three conditions. In the case of the above example *Imitators* was used in the animated, *The Little Kangaroo* in the static and *The Bear is in Love with Butterfly* in the control condition. The order of the animated and static condition was the opposite for half of the participants who started with the static book. The order of the story retelling and vocabulary post-tests was different for half of the children who started with the vocabulary tests. The order of the books retold was random. The order of the four vocabulary tests was fixed and the same for all children.*

Post-testing on the second day conducted right after the third session included a retelling of the two stories that they had heard three times and the control story, which they did not hear. The order of the books retold was random. We also tested knowledge of 9 non-words, three from each story. We used four vocabulary tests assessing different levels of word knowledge. The order of the four vocabulary tests was the same for all children, starting with the expressive tests in order to avoid any possible learning from the receptive tests. The order of the retellings and the vocabulary tests was counterbalanced: 19 children started with the story retelling, while 20 children completed the vocabulary tests first. See **Table 1** for an example schedule.

### Materials

Three animated storybooks [The Little Kangaroo (van Genechten, 2009), Imitators (Veldkamp, 2006), Bear is in Love with Butterfly (van Haeringen, 2004)] were used in the experiment. In each book three verbs were substituted by non-words (see Appendix for the list of target words). The word ‘jumping’ [‘springen’], for instance, was replaced by the non-word ‘trinnen’; see **Figure 1**. Each of the three books included three non-words, which resulted in nine non-existing target words. In each story two of three non-words were mentioned twice in the oral text and one once. A female adult recorded the narration.

All three stories were animated by the same company^1^. To make the static illustrations similar to the animated illustrations, we selected the most representative still frame of each scene in the animated books and presented this for exactly the same amount of time as the animated scene; see in the first row of **Figure 1** the illustration that was presented in the static condition and a series of screen dumps from the animated version in the second row. There was some slight variation between the three books: Bear is in Love with Butterfly included 397 words, The Little Kangaroo 516 words, and Imitators 509 words. Accordingly, the duration of the readings were somewhat different too: to read Bear is in Love with Butterfly took 194 s, The Little Kangaroo 232 s, and Imitators 252 s. We corrected for differences in length of presentation by dividing fixation durations for the whole book by the duration of the stories.

### Apparatus

While the recorded voice read the stories to the children their eye movements within the static and animated illustrations were recorded with a Tobii T120 eye tracker with a screen resolution of 1280 × 1024. The eye positions were assessed 120 times per second (120 Hz) by illuminating both eyes with infrared LED and measuring the reflected light. The system has a high accuracy (<0.5 cm) and allows for some head movement, typically resulting in a temporary accuracy error of approximately 0.2 visual degrees. For fast head movements (i.e., over 25 cm/s), there is a 300-ms recovery period to full tracking ability (Timing Guide for Tobii Eye-Trackers and Eye-Tracking Software, 2010). The eye tracker was paired with a laptop computer with a screen resolution of 1024 × 768. To guarantee the most optimal registration of eye movements, participants were seated at a distance of 60–70 cm from the eye tracker. At the start of each session, the eye tracker was calibrated for each participant: Children were asked to fixate five dots that appeared in different positions on a screen with a background color similar to the background color of the stimuli. The procedure did not require any effort on the part of the child and took at most 120 s.

### Measures

#### Visual Attention at the Illustrations

The total fixation time on the illustrations in a storybook was calculated and divided by the duration of rendering the complete book because there were some variations in the total lengths of the three stories. Additionally, we assessed the number of fixations per storybook and calculated the average duration of fixations while looking at the storybooks. This was done for all three sessions for both books in the two experimental conditions.

Furthermore, per book three illustrations were chosen for detailed eye-tracking analyses. We chose illustrations that depicted the non-words. This detail of the illustration was in motion in the animated condition and clearly visualized in the static condition; see as an example the director in the scene depicted in **Figure 1**. The details were the same size in the animated and static condition and the animated illustration did not include additional information as compared to the still illustration. In **Figure 1**, for instance, the still picture also shows a jumping director.

We defined details that visualized the non-words as Areas of Interest (AOIs) using the software of Tobii Studio 2.2.6. Eye-movement data (i.e., the time that children fixated the AOI and the number of fixations within the AOI) was scored using Tobii Studio’s fixation filter with the default settings for velocity and distance threshold (Tobii Technology, 2010 User Manual: Tobii Studio, 2010). We divided the time that children fixated the AOI in an illustration by the time that they looked at the whole illustration in order to control for any differences in the time children looked at the screen between the conditions. This was done for the three AOIs per book. The average percentage was calculated as an indicator for each condition and each session. Additionally, we divided children’s fixation duration at an AOI by the number of fixations as an indicator of average fixation duration. The average fixation duration was also calculated for each condition and session.

For four children data quality was low, i.e., eye movements were registered for less than 50% of the time during at least one session in one of the conditions. Due to low data quality, these children’s fixation times were extremely low. For the eye-tracking analyses these four children were excluded and, accordingly, data of 35 children were used. Additionally, outlying scores were winsorized in order to normalize the distribution of the scores. In all, 20 scores (2.3%).

#### Story Retelling

Resembling the common activity of independently “reading” a familiar storybook, we asked children to retell the three stories using the static illustrations of the stories (cf. Sulzby, 1985; Korat et al., 2014). Pictures were available during the retelling to prevent that children confine themselves to a brief summary and to enable a retelling of the control story. The experimenter instructed the children as follows: “Last time you listened to stories. Now I would like you to tell me stories.” When the story was unfamiliar to the child because it was in the control condition, the examiner said, “Here are the pictures of a story. Please tell me this story.” Thus, results of the control condition show how much information children can figure out based solely on the pictures without ever listening to the story making the difference between the experimental conditions and the control reflect effects of book reading sessions. The experimenter asked general questions when children stopped talking like ‘What is happening here?’ or ‘Who is this?’. Children’s retellings were transcribed and the transcripts were checked with the video recording of the session by another coder.

We coded how many content words from the original story appeared in the retellings of the stories, in addition to the percentage of correctly summarized pages in children’s retellings. According Sulzby’s (1985) scale of reading a favorite book the amount of verbally reproduced text is an important indicator of children’s level of story comprehension. According to previous findings (de Jong and Bus, 2002) this variable was a better indicator of children’s story comprehension than coding the similarity between the story told and the original story probably because this is a more subjective measure. We also coded whether or not the non-words were used in the story retellings in order to learn how often children learn the new words they were taught (cf. Korat et al., 2014). One child refused to retell the stories so analyses regarding story comprehension were conducted on the data of 38 children. Inter-rater reliabilities for verbally reproduced text, the similarity between the retold and the original story, and the use of non-words in the retellings were high. Intraclass correlation coefficients equaled 0.94, 0.99, and 1.00, respectively.

#### Vocabulary Tests

Familiarity with the nine target non-words was assessed with four tests measuring receptive and expressive knowledge of the words. We started with the two expressive vocabulary tests in order to avoid learning from the receptive tests in which children heard the target words.

##### Expressive vocabulary test

**FIGURE 2:**
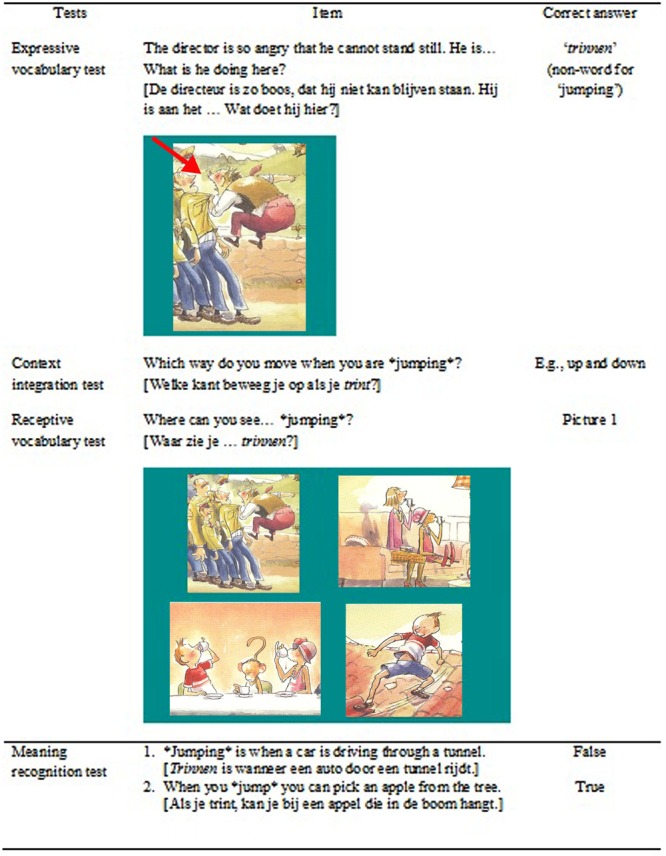
**Examples of the four vocabulary tests assessing knowledge of the non-word ‘trinnen’ which stands for the word ‘jumping’ including still frames from the animated version.** Copyright 2014 by Het Woeste Woud.

The expressive vocabulary test measured whether children could use the word in the context of the storybooks. With the corresponding illustration on the screen children were asked to complete a sentence with the non-word missing. Sentences were phrased differently than in the stories. Only answers including the target word scored 1, any other answers 0. See **Figure 2** for an example. Item-level inter-rater reliability was excellent (average κ = 1.00). Only one child used any of the target words so no further statistical analyses were conducted on this measure.

##### Context integration test

The context integration test assessed whether children could make sense of the target words in a novel context. It used open-ended questions prompting an expressive explanation of the target word (e.g., “Which way do you move when you are ^∗^jumping^∗^?”). Only answers with information reflecting the meaning of the target word were awarded 1 (e.g., “You go up and down”), any other answers (e.g., ‘to the right’) were scored 0. Item-level inter-rater reliability was good (average κ = 0.89). Children rarely gave good answers resulting in a much skewed outcome. Thus, statistical analyses were not conducted on this measure.

##### Receptive vocabulary test

The receptive vocabulary test measured children’s understanding of the target word in the context of the storybooks. This test was a multiple-choice test where children had to choose the corresponding picture from 4 options. Target pictures and distractors were chosen from the same storybooks. See **Figure 2** for an example. Item-level inter-rater reliability was good (average κ = 0.78). More than half of the children performed above chance level (25%) in both the animated (*p* < 0.001) and the static condition (*p* < 0.001). This was not the case in the control condition (*p* = 0.34).

##### Meaning recognition test

The meaning recognition test assessed word comprehension independent of the context of the storybooks. Using two yes/no questions per word regarding the meaning of the word presented in a quasi-random order, children’s receptive transfer knowledge of the non-words was tested. See **Figure 2** for an example. Item-level inter-rater reliability was good (average κ = 0.72). Children did not perform above chance level in any of the conditions (animated: *p* = 0.75, static: *p* = 0.52, control: *p* = 0.20) so scores on this test were not further analyzed.

## Results

### Story Comprehension

We carried out two ANOVAs with repeated measures for both indicators of story comprehension: children’s recall of the language (recall of content words) and the content of the story (the similarity between children’s stories and the original stories). As we found previously (de Jong and Bus, 2002) correlations between these two measures were rather high; animated condition: *r*(38) = 0.63, *p* < 0.001; static condition: *r* (38) = 0.79, *p* < 0.001. Two planned contrasts were conducted regarding the effects of condition: (1) between the experimental and the control conditions to test the effect of book reading, and (2) between the animated and the static conditions. Because students were grouped within schools, even a weak school effect (intraclass correlation) can substantially deflate variation within schools. To control for such differences school was entered as a between-subject factor in the ANOVA. An ANOVA with repeated measures for recall of the story language per condition and school (school 1, 2, and 3) as a between-subject factor was carried out.

Children recalled more from the language of the animated and the static stories as compared to the control condition [*F*(1,35) = 60.45, *p* < 0.001, η_p_^2^ = 0.63] showing that children could not guess the story language by just looking at the pictures. See **Table 2** for descriptive statistics. More importantly, children recalled significantly more content words from the animated as compared to the static condition [*F*(1,35) = 5.87, *p* = 0.02, η_p_^2^ = 0.14] showing an advantage of animations. There was no main effect of school nor did it have a significant interaction effect with condition.

**Table 2:** Descriptive statistics on the outcome measures of story retelling and receptive vocabulary for each condition.

	Animated condition *M* (*SD*)	Static condition *M* (*SD*)	Control condition *M* (*SD*)
Story recall (number of content words recalled)	12.74 (5.75)	11.40 (6.74)	2.95 (1.86)
school 1 school 2 school 3	11.89 (4.73) 11.80 (4.49) 13.25 (6.41)	9.44 (4.85) 8.80 (4.87) 12.67 (7.49)	3.22 (1.72) 3.40 (2.88) 2.75 (1.73)
Story recall (percentage of pages correctly summarized) school 1 school 2 school 3	67.58 (23.67) 71.33 (15.44) 61.85 (16.77) 67.36 (27.51)	61.18 (27.94) 49.14 (26.14) 54.70 (33.81) 67.04 (26.79)	4.10 (9.44) 0.69 (2.08) 3.33 (7.45) 5.46 (11.20)
Receptive vocabulary test (number of correctly identified items out of three) school 1 school 2 school 3	1.51 (0.79) 1.22 (0.83) 1.83 (0.75) 1.54 (0.78)	1.36 (0.81) 0.78 (0.83) 2.00 (0.63) 1.42 (0.72)	0.95 (0.92) 0.67 (0.87) 1.33 (1.21) 0.96 (0.86)

We tested a similar model for the similarity between children’s stories and the original stories. From the 38 children thirty scored 0 in the control condition so this variable was non-normally distributed and thus we could not include the first contrast between control and experimental conditions in the ANOVA. However, it is obvious that children recalled more similar stories in the experimental conditions (animated: *M* = 67.6%, *SD* = 23.67, static: *M* = 61.2%, *SD* = 27.94) than in the control condition (*M* = 4.1%, *SD* = 9.44). The contrast between animated and static condition was significant: children’s recollection of the stories was more similar in the animated as compared to the static condition [*F*(1,35) = 5.51, *p* = 0.03, η_p_^2^ = 0.14). There was no main effect of school. There was, however, a significant interaction effect between school and condition [*F*(2,35) = 3.46, *p* = 0.04, η_p_^2^ = 0.17), meaning that there was a difference between the animated and static conditions for two out of three schools.

### Word Learning

Only three out of 38 children used non-words from the story in the retelling, one in the animated and two in the static condition. Thus, the effect of condition could not be tested on this variable.

Of the four words learning tests children only showed learning in the receptive vocabulary test. Scores on the other three tests showed bottom effects. We carried out an ANOVA with repeated measures for the receptive vocabulary test in the three conditions (animated, static, control) and school (school 1, 2, and 3) as a between-subject factor. Two planned contrasts were conducted regarding the effects of condition: 1. between the intervention and the control conditions, and 2. between the animated and the static conditions. There was an effect of book reading on receptive word learning: children performed significantly better in the animated and the static conditions as compared to the control condition [*F*(1,36) = 5.76, *p* = 0.02, η_p_^2^ = 0.14]. See **Table 2** for descriptive statistics. However, there was no significant difference between the animated and static conditions [*F*(1,36) = 0.44, *p* = 0.51, η_p_^2^ = 0.01]. There was no main effect of school nor did it have a significant interaction effect with condition.

### Visual Attention to All Illustrations in the Books

We carried out an ANOVA with repeated measures for the percentage of total time that it took to read the book in which children fixated the illustrations of the books. Within subject factors were condition (animated versus static) and session number (first, second, or third). We carried out two planned contrasts for session number: the contrast between the first and the second and between the first and the third session in order to test whether attention to the illustrations decreased over sessions. School (school 1, 2 and 3) was entered as between-subject factor.

We found a significant main effect of condition on percentage fixations [*F*(1,32) = 19.86, *p* < 0.001, η_p_^2^ = 0.38], meaning that children attended the screen more in the animated as compared to the static condition. Contrasts showed that children attended the screen less during the second session as compared to the first [*F*(1,32) = 18.56, *p* < 0.001, η_p_^2^ = 0.37] but visual attention was similar in the third session as compared to the first [*F*(1,32) = 0.04, *p* = 0.84, η_p_^2^ = 0.001]. There was no main effect of school nor did it have a significant interaction effect with condition. See **Table 3** for descriptive statistics.

**Table 3:** Visual attention to the illustrations in animated and static books during the first, second, and third session.

	Total fixation time on the illustrations, corrected for the length of the book (in percentages)^a^		Average fixation duration on the illustrations (in seconds)	
	Animated condition *M* (*SD*)	Static condition *M* (*SD*)	Animated condition *M* (*SD*)	Static condition *M* (*SD*)
Session 1	0.84 (0.07)	0.79 (0.07)	0.37 s (0.09)	0.34 s (0.07)
school 1	0.84 (0.09)	0.82 (0.05)	0.36 s (0.11)	0.36 s (0.04)
school 2	0.88 (0.03)	0.81 (0.03)	0.39 s (0.06)	0.36 s (0.10)
school 3	0.84 (0.07)	0.78 (0.08)	0.37 s (0.09)	0.33 s (0.07)
Session 2	0.82 (0.08)	0.77 (0.09)	0.38 s (0.09)	0.34 s (0.08)
school 1	0.85 (0.04)	0.79 (0.08)	0.41 s (0.07)	0.37 s (0.05)
school 2	0.77 (0.10)	0.73 (0.08)	0.40 s (0.07)	0.33 s (0.08)
school 3	0.82 (0.08)	0.76 (0.10)	0.37 s (0.10)	0.33 s (0.08)
Session 3	0.85 (0.06)	0.80 (0.07)	0.40 s (0.08)	0.36 s (0.06)
school 1	0.86 (0.05)	0.82 (0.08)	0.41 s (0.06)	0.39 s (0.05)
school 2	0.85 (0.09)	0.81 (0.06)	0.40 s (0.06)	0.35 s (0.05)
school 3	0.85 (0.06)	0.80 (0.07)	0.40 s (0.09)	0.35 s (0.07)

*^a^Percentage score, children’s total fixation time at the illustrations were divided by the presentation time of the book in order to correct for the slight differences between the books.*

The same analysis was applied to the average duration of fixations on the illustrations. We found a main effect of condition [*F*(1,32) = 5.64, *p* = 0.02, η_p_^2^ = 0.015], showing that fixations on the illustrations in the animated books were longer as compared to the fixations on illustrations in static books. There was no difference between the first and the second session [*F*(1,32) = 1.31, *p* = 0.26, η_p_^2^ = 0.04]. However, children had significantly longer average fixations on the third as compared to the first session [*F*(1,32) = 5.95, *p* = 0.02, η_p_^2^ = 0.16]. There was no main effect of school nor did it have a significant interaction effect with condition.

### Attention to Motion While Looking at the Target Illustrations

We carried out an ANOVA with repeated measures for the percentage of total fixation time spent on the selected detail (in motion in the animated version and a still detail in the static version). With Tobii Studio software (version 2.2.6) we selected the same areas in both versions of the books and calculated fixation durations on these target details (AOI). These scores were divided by children’s fixation duration on the whole illustration in order to control for overall elevated attention to animated illustrations. Within-subject factors were condition (animated versus static) and session number (1, 2, and 3). Between-subject factor was school (school 1, 2, and 3). The ANOVA resulted in a significant main effect of condition [*F*(1,32) = 19.16, *p* < 0.001, η_p_^2^ = 0.38] indicating that children focused more on the details when they were in motion. As is shown in **Table 4**, in the static condition children fixated 69% of the time on the target detail that was highlighted in the text. In the animated condition the score was 9% higher. The contrast between the first and the third session was not significant [*F*(1,32) = 2.72, *p* = 0.11, η_p_^2^ = 0.08]. However, children were more attentive to the AOIs during the first as compared to the second session [*F*(1,32) = 8.05, *p* < 0.01, η_p_^2^ = 0.20]. There was no main effect of school nor did it have a significant interaction effect with condition.

**Table 4:** Visual attention to the salient movement depicting non-words in the animated and static condition per session.

	Fixation time on AOIs (in percentages)		Average duration of fixations while looking at AOIs (in seconds)	
	Animated condition *M* (*SD*)	Static condition *M* (*SD*)	Animated condition *M* (*SD*)	Static condition *M* (*SD*)
Session 1	0.80 (0.08)	0.70 (0.12)	0.38 s (0.11)	0.31 s (0.07)
school 1	0.79 (0.11)	0.69 (0.12)	0.35 s (0.14)	0.31 s (0.03)
school 2	0.86 (0.08)	0.70 (0.03)	0.45 s (0.06)	0.34 s (0.08)
school 3	0.79 (0.08)	0.70 (0.13)	0.37 s (0.11)	0.30 s (0.07)
Session 2	0.76 (0.11)	0.68 (0.12)	0.38 s (0.10)	0.30 s (0.08)
school 1	0.77 (0.13)	0.67 (0.15)	0.39 s (0.07)	0.33 s (0.10)
school 2	0.77 (0.13)	0.56 (0.09)	0.42 s (0.10)	0.28 s (0.10)
school 3	0.76 (0.10)	0.70 (0.10)	0.37 s (0.10)	0.30 s (0.07)
Session 3	0.77 (0.12)	0.69 (0.11)	0.41 s (0.12)	0.32 s (0.06)
school 1	0.78 (0.16)	0.67 (0.12)	0.40 s (0.05)	0.33 s (0.03)
school 2	0.73 (0.12)	0.70 (0.11)	0.43 s (0.14)	0.30 s (0.08)
school 3	0.78 (0.10)	0.70 (0.11)	0.41 (0.13)	0.32 s (0.06)

The same model was applied to children’s average fixation duration while looking at the selected details. There was a significant main effect of condition [*F*(1,32) = 23.92, *p* < 0.001, η_p_^2^ = 0.44], showing that children’s average fixations were longer on the moving details in the animated condition as compared to the same details in the static book. Average fixation durations for the first session were not different from the second [*F*(1,32) = 0.14, *p* = 0.71, η_p_^2^ = 0.004] or the third session [*F*(1,32) = 0.97, *p* = 0.33, η_p_^2^ = 0.03]. There was no main effect of school nor did it have a significant interaction effect with condition.

## Discussion

### Learning Form Animated Stories

The effects of animated illustrations on children’s story recall, word learning and visual attention during storybook reading were investigated in the present study. We found, in line with the results of a recent meta-analysis (Takacs et al., 2015), that children recalled significantly more story language and recollected more similar stories to the original stories with the help of animated illustrations. Since there were, unlike previous studies (e.g., Verhallen et al., 2006) no other multimedia additions like sounds and music in the stories in the present study but only motion in the illustrations, the current findings corroborate the hypothesis that motion can elevate story comprehension and thus is a crucial part of a well-designed multimedia environment for children’s storybooks (cf. Takacs et al., 2015). This is the first study, to our knowledge, that shows the effects of animated books that include motion alone, and no music or sounds, on children’s story comprehension. We found a medium effect size (η_p_^2^ = 0.14), similar to the advantage found for multimedia stories as compared to print storybooks in a previous meta-analysis (Takacs et al., 2015). Further studies are needed to investigate any separate effects of other multimedia features like sound and music to create clear guidelines for designers of multimedia stories.

In contrast to the second hypothesis, there was no difference between the animated and the static conditions in terms of word learning. However, the results do support children acquired word knowledge from book reading. The vocabulary results show that after three repeated book readings children had elementary knowledge of completely novel words (non-words). Children showed significant word learning on a receptive level. On the expressive level there were no effects. The fact that children did not learn more from animated than from static books might be because learning did not go beyond the earliest phase of word learning: understanding a word in the context in which children encountered it previously. Transfer of this knowledge to other contexts and expressive use of the word seem to come later on with repeated exposures to the word (Nagy and Scott, 2000). Similar to prior findings, we may expect that especially the step from receptive to expressive knowledge with repeated exposure is facilitated by animated books (Smeets and Bus, 2012).

### Different Processing Strategies

Most interestingly, the fine-grained results of the current study evidence that, children use different processing strategies while looking at animated and static illustrations. In line with previous results (Evans and Saint-Aubin, 2005; Verhallen and Bus, 2011), children paid most visual attention to details in pictures that are simultaneously highlighted in the story text. When visual details in the pictures were in motion they received even more visual attention as compared to the same details in the static illustrations. We actually found that the duration of fixations on details depicting the core of the story was longer on average in the animated condition despite that the animated pictures were not more informative. Animated pictures may, as a result of a longer and steadier focus on details that are highlighted in the text, facilitate dual coding of verbal and non-verbal information and this may explain why we find better understanding of animated stories again and again (Takacs et al., 2015). Furthermore, children’s eyes were moving less between the different visual elements of the animated illustration and focused more on particular details. That is, children were less inclined to explore the whole picture by jumping to different visual elements but fixated more on the most important details. The longer average fixations might reflect deeper processing of the relevant details in the illustrations (Rayner, 2009). In other words, there is strong support for the hypothesis that, due to motion, children’s visual attention is longer and more steadily focused on details that are highlighted by the story text resulting in more in-depth exploration of those details in the illustration and probably better integration of the verbal and non-verbal information. In sum, motion seems to scaffold learning by guiding children’s visual attention.

There is also evidence that children attended the screen for a longer time in total and looked away from the screen less when the illustrations were animated. The most plausible explanation is that this is a side effect of focusing longer on particular details in motion while exploring the picture. We did, however, control for this elevated overall attention in the analyses regarding fixations on the highlighted details. These analyses showed that, regardless of overall attention, there was a difference in processing the most important details when they were in motion and when they were static. Although the overall findings for processing visual information indicate a different processing strategy as a result of motion, we cannot entirely exclude that comprehension in the animated condition as compared to the static condition also improved as a result of higher attention as may be indicated by children’s longer fixations on the whole book. That is, listening to the animated version of the storybooks may be more engaging than listening to the static version and this is, in addition to a different processing strategy, an incentive as well for better story understanding.

An unexpected result of the present study was the effect of session number on children’s visual attention during the stories; that is, children were less attentive to illustrations of the stories and the moving details in the illustrations on the second repetition in both conditions. This is most probably due to the fact that the first session was conducted at the same day as the second. Children encountered the same story a couple of minutes earlier which may explain why their attention dropped. However, children were similarly attentive to the illustrations and the motion in the animations during the third repetition as they were on the first occasion in both conditions.

### Limitations

The non-words in the present study were inserted in place of mostly high-frequency verbs like jumping that children are likely to understand and use. Thus, children might have not been motivated to use the novel words when retelling the story or completing sentences in the expressive vocabulary test because they already knew a word for these actions. It is plausible that children’s expressive word knowledge was thus underestimated in the present study. It might be better to investigate novel word learning in the context of novel actions and phenomena for which children do not yet have labels in order to better estimate expressive learning of the words.

Another limitation was the use of the static illustrations of the stories in the retellings of both the static and the animated conditions as well as in the vocabulary tests. This was decided in order for the experimenter not is influenced by the condition when interacting with the child during retelling the story. However, this might have underestimated the effects of animated illustrations on children’s recall and word learning because the same animated pictures as seen during the story might have prompted more extensive recalls and better performance on a multiple-choice test based on the illustrations of the storybook like the receptive vocabulary test in the present study.

## Conclusion and Suggestions for Future Studies

In sum, motion attracts visual attention and changes the way children process illustrations. Animated illustrations that are closely matched to the story text have more potential to direct children’s attention to specific details of the picture as compared to static illustrations and when details in motion are simultaneously highlighted by the text animation may promote dual-coding (Paivio, 2007). We expect that the focus on motion in pictures explains why children look longer overall at the illustrations of animated books as compared to static books although we cannot exclude that they are also more alert when illustrations are animated. Although children are attracted to animations and specifically to motion in the animations, they do not seem to be mesmerized by the visual stimuli. On the contrary, they benefit from more intensive visual stimuli.

These findings have important practical implications for designers and developers of electronic storybooks and for caretakers and teachers navigating on the market of children’s electronic storybooks. Animations and motion seem to be a powerful tool to guide children’s visual attention to particular details that are meaningful from the story’s point of view. It seems most plausible that well-designed animations will focus children’s attention on the parts of the illustrations that depict the text of the story thus facilitating the integration of verbal information in the story and the non-verbal stimuli of animation and children’s story comprehension. Consequently, high-quality electronic storybooks will utilize the benefits of animations and other multimedia features creating congruency between the story text and the technological elements like animations. By thus guiding children’s visual attention (visual scaffolding) multimedia books may reinforce the beneficial effects of storybook reading. If our finding for animation and motion can be replicated in future studies us can more confidently advise developers and customers of electronic storybooks.

For designers of storybook apps it is also important to realize that motion in pictures is helpful when the animation indeed depicts the language of the narration. We hypothesize that animation that have only decorative purposes as we often see in storybook apps may not add to children’s story comprehension and might even interfere with learning. Such incidental animations with no direct connection to the text of the story are hypothesized to distract them from the story by posing a high cognitive load on their working memory. This hypothesis is in line with a meta-analytic finding of Höﬄer and Leutner (2007), showing no additional benefit of decorative animations for adults’ learning as compared to a moderate effect of representational animations. Future research should test this hypothesis.

## Author Contributions

All authors listed, have made substantial, direct and intellectual contribution to the work, and approved it for publication.

## Conflict of Interest Statement

The authors declare that the research was conducted in the absence of any commercial or financial relationships that could be construed as a potential conflict of interest.

## Footnotes

1 http://hetwoestewoud.nl
