# Supplementary material for: Benefits of Motion in Animated Storybooks for Children’s Visual Attention and Story Comprehension. An Eye-Tracking Study
Source: Front Psychol. 2016 Oct 13;7:1591. doi: 10.3389/fpsyg.2016.01591 (PMC5062825; doi:10.3389/fpsyg.2016.01591)
Supplement: Supplementary file 1 [file Data_Sheet_1.docx]

Appendix

List of target words per book

| Book | Original word (verb) | Non-word used instead of the word | The number of times the non-word mentioned in the text |
| --- | --- | --- | --- |
| *Bear is in Love with Butterfly* |  |  |  |
|  | to write (schrijven) | ‘drimmelen’ | 2 |
|  | to play (spelen) | ‘beteenen’ | 2 |
|  | to fan (aanwakkeren) | ‘blukkeren’ | 1 |
| *Imitators* |  |  |  |
|  | to walk (lopen) | ‘aaften’ | 2 |
|  | to jump (springen) | ‘trinnen’ | 2 |
|  | to bang (bonken) | ‘tronkten’ | 1 |
| *The Little Kangaroo* |  |  |  |
|  | to flutter (fladderen) | ‘zoofen’ | 2 |
|  | to run (rennen) | ‘pirten’ | 2 |
|  | to follow (volgen) | ‘goegen’ | 1 |
